# Evolution takes multiple paths to evolvability when facing environmental change

**DOI:** 10.1073/pnas.2413930121

**Published:** 2024-12-31

**Authors:** Bhaskar Kumawat, Alexander Lalejini, Monica M. Acosta, Luis Zaman

**Affiliations:** ^a^Department of Ecology and Evolutionary Biology, University of Michigan, Ann Arbor, MI 48109; ^b^Center for the Study of Complex Systems, University of Michigan, Ann Arbor, MI 48109; ^c^School of Computing, Grand Valley State University, Allendale, MI 49401

**Keywords:** evolvability, mutation rate, digital evolution, fitness landscape

## Abstract

Life at all scales is surprisingly effective at exploiting new opportunities, as demonstrated by the rapid emergence of antimicrobial resistance and novel pathogens. How populations acquire this level of evolvability and the various ways it aids survival are major open questions with direct implications for human health. Here, we use digital evolution to show that changing environments facilitate the simultaneous evolution of high mutation rates and a distribution of mutational effects skewed toward beneficial phenotypes. The evolved mutational neighborhoods allow rapid adaptation to previously encountered environments, whereas higher mutation rates aid adaptation to completely new environmental conditions. By precisely tracking evolving lineages and the phenotypes of their mutants, we show that evolving populations localize on phenotypic boundaries between distinct regions of genotype space. Our results demonstrate how evolution shapes multiple determinants of evolvability concurrently, fine-tuning a population’s adaptive responses to unpredictable or recurrent environmental shifts.

## Significance

That all the diversity of life constitutes what Erasmus Darwin called “a single living filament”—an unbroken chain of descent from the last universal common ancestor—is evidence of life’s fundamental adaptability. However, the evolutionary processes that shape this ability to adapt (evolvability) remain elusive because of the required resolution and timespan of observations. Using evolving, self-replicating computer programs, we find that multiple pathways to increased evolvability emerge concurrently and distinctly aid adaptation. One pathway (evolved mutational landscapes) allows rapid adaptation to previously seen environments, while the other (higher mutation rates) allows rapid adaptation to entirely new environments. This multifaceted picture of evolvability helps us understand how organisms deal with ever-changing conditions and relentlessly explore nature’s opportunities for innovation.

Life has flourished in seemingly uninhabitable parts of the world, and even as ongoing environmental change challenges ecosystems, rapid adaptation is a frequent response of populations in nature and in the lab (1–4). Emerging viral pathogens like SARS-CoV-2 and avian influenza (H5N1) are notable examples of pathogens that have shown a remarkable capacity to adapt to changing host immunity (5–9). Similarly, controlling microbial growth is a constant struggle across industries—for example, in wastewater treatment, healthcare, and agriculture—precisely because antimicrobial resistance evolves so readily (10–12). However, this ability to evolve, or evolvability, is rarely a direct target for natural selection since these traits do not typically translate into higher fitness. Yet rapid adaptation is readily seen in nature during range expansions into new environments and in populations facing environmental fluctuations (3, 4, 13, 14). In the lab, directed evolution has shown that second-order selection for evolvability does exist, though an understanding of the mechanisms involved has only recently begun to emerge (15–18). It is unclear why evolution consistently produces populations that evolve so relentlessly. So far, evidence of increased evolvability has generally relied on indirect measures. Direct observation of the evolution of evolvability is intractable due to the timescales and resolution of historical data that are required and presents a major challenge to addressing this question (19–23). A holistic picture of the various processes that drive the evolution of evolvability is necessary to understand why evolution is so effective and to limit (or harness) its future potential (24).

At its core, Darwinian evolution is fueled by the continual emergence and spread of new phenotypic variants in a population, which can emerge as a result of multiple coupled processes (25). Replication of genetic material during the reproduction of an organism is susceptible to occasional errors that lead to mutations with a frequency quantified by the mutation rate (measured per generation). However, only a small proportion of errors result in adaptive phenotypic change, since most mutations are neutral or deleterious (26). The set of mutants accessible from existing genotypes in a population and their corresponding phenotypes (i.e., the mutational neighborhood) specifies the fraction of mutations that are ultimately beneficial (27). Collectively, the mutation rate and mutational neighborhood govern the pace at which populations generate new phenotypes, providing the raw materials on which natural selection can act (22). Thus, we hypothesized that these two coupled processes would be implicated in an escalation in evolvability.

Previous computational work has shown that a tendency to mutate toward phenotypes adapted to historically encountered environments can evolve as a result of fluctuating selection (28–32). In contrast to this limited definition, evolvability observed in macroevolution is regarded as a latent property of entire clades that promotes phenotyptic divergence irrespective of any selective advantage it provides (33, 34). Similarly, work on mutators and mutational modifiers focuses on an escalation in the rate at which variation is created, but ignores the phenotypic identity of particular variants (35–37). Bridging these two scales, recent work analyzing fossil data suggests that microevolutionary evolvability shapes the rate of macroevolutionary diversification because the gradient of selection is constantly being perturbed as environments shift (38). Like many ideas in evolutionary biology, evolvability is susceptible to the overuse of adaptationism, and the abundance of different definitions in the literature likely adds to the confusion (20, 39–41). Evolvability is thus used ambiguously to describe several distinct properties and may arise through distinct pathways. The conditions that shape the simultaneous evolution of multiple determinants of evolvability, how these determinants influence each other, and the way they aid adaptation in recurring or novel environments remain unexplored (23).

**Fig. 1.**
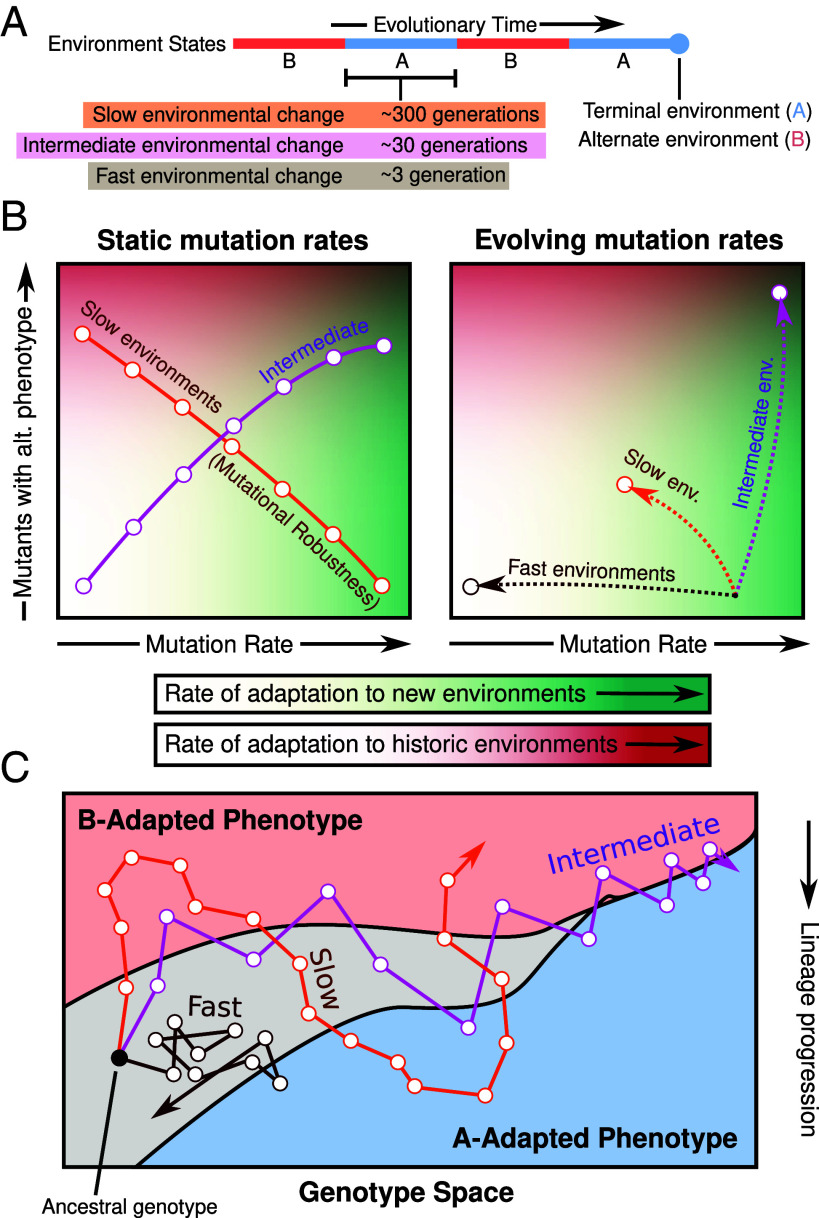
Key results for three environments. (*A*) We study evolution under environments that switch between two states (Blue = A and Red = B) at three different rates (Orange = Slow, Purple = Intermediate, Brown = Fast). If evolution concludes in state A, B is referred to as the “alternate” environment, and vice versa. Environments switch approximately every 3, 30, or 300 generations. (*B*) We perform two types of experiments, those where the mutation rate is fixed (*Left*) and those where the mutation rates are allowed to evolve (*Right*). When mutation rates are fixed and the environment switches slowly (orange curve), we observe mutational robustness, where increasing mutation rates lead to a decrease in the number of mutants with an alternate phenotype. However, at intermediate rates (purple curve), increasing mutation rate favor alternate phenotype mutants in the mutational neighborhood. When mutation rates evolve, the intermediate environments lead to high mutation rates as well as a large number of alternative phenotypes in the mutational neighborhood. We find that while elevated mutation rates help adapt to completely new environments (green shade), an increasing number of alternate phenotype mutants allows for quicker adaptation to historic environments (red shade). Thus, intermediate environments promote evolution of evolvability through two different pathways and buffer the population against different types of environmental change. (*C*) This multifaceted increase in evolvability can be partly explained by the localization of evolving lineages on boundaries between phenotypic regions in the genotype space. Here, this localization is shown as the intermediate lineage (purple) zig-zagging across the boundary between A and B-adapted phenotypes.

In this work, we use populations of self-replicating computer programs in the digital evolution framework Avida to study how environmental conditions influence the evolution of evolvability (42). We measure evolvability both indirectly (in terms of changes to the mutation rate and mutational neighborhood) and directly by measuring the rate of adaptation to new and historic environments. The computational nature of this systems affords us unparalleled precision in tracking the mutational changes that occur along lineages over evolutionary time, while recording information about the external environment when those mutations arise. It also allows an in-depth analysis of the local mutational landscapes of these lineages, examining up to two mutational steps and encompassing over three million mutants per genotype. Using this framework, we study the mutation rates and structure of the mutational neighborhoods that evolve as a result of exposure to different treatments (Fig. 1*A*). We assess the performance of evolved organisms in their native and nonnative conditions to highlight the way these determinants shape future adaptation. Our findings demonstrate the existence of specific modes of environment change where the mutation rate and mutational neighborhoods evolve in ways that enhance further evolutionary innovation synergistically (Fig. 1*B*). Finally, the evolutionary dynamics show signatures of an insufficiently explored nonequilibrium evolutionary process that captures evolving lineages on boundaries between phenotypes in the space of genotypes (Fig. 1*C*).

## Evolution in Avida

Organisms in Avida are autonomous, asexual, self-replicating computer programs that compete for space and execution time in a virtual environment (42, 43). These organisms possess a genome—a linear sequence of computer instructions—that enables them to reproduce and perform tasks (*SI Appendix*, Fig. S1*A*). Each task is an arithmetic/logical function that requires the execution of multiple, well-ordered instructions in the genome to be completed. As such, individual instructions do not have direct fitness consequences, except when they are associated with groups of instructions that perform tasks. For example, the “AND” task requires at least nine functional instructions be performed in order (*SI Appendix*, Table S1). The Avida instruction set includes operations for basic numerical manipulations, flow control (e.g., conditional logic and looping), input, output, and the building blocks of self-replication. To reproduce, organisms must duplicate their genome instruction-by-instruction and then divide, using a copy-loop that is also encoded in their genome. Copy operations are imperfect and can result in mutations to the instructions being written to the offspring genome. These mutations provide the de novo variation required for evolution. Time in Avida is measured in updates (average generation time of an organism ∼10 updates). After division, new offspring are placed randomly in the world, replacing any existing occupants. Organisms thus compete for space, which drives selection to improve their replication rate in order to not be overwritten by competitors. Performing tasks allows organisms to acquire more compute cycles (determined by a merit multiplier) from the Avida scheduler, which increases their rate of execution, and indirectly (and nonlinearly) increases their rate of replication. Increases in fitness are thus not assigned but can only be measured empirically as the outcome of organisms interacting with their environment (*SI Appendix*, Fig. S2).

**Fig. 2.**
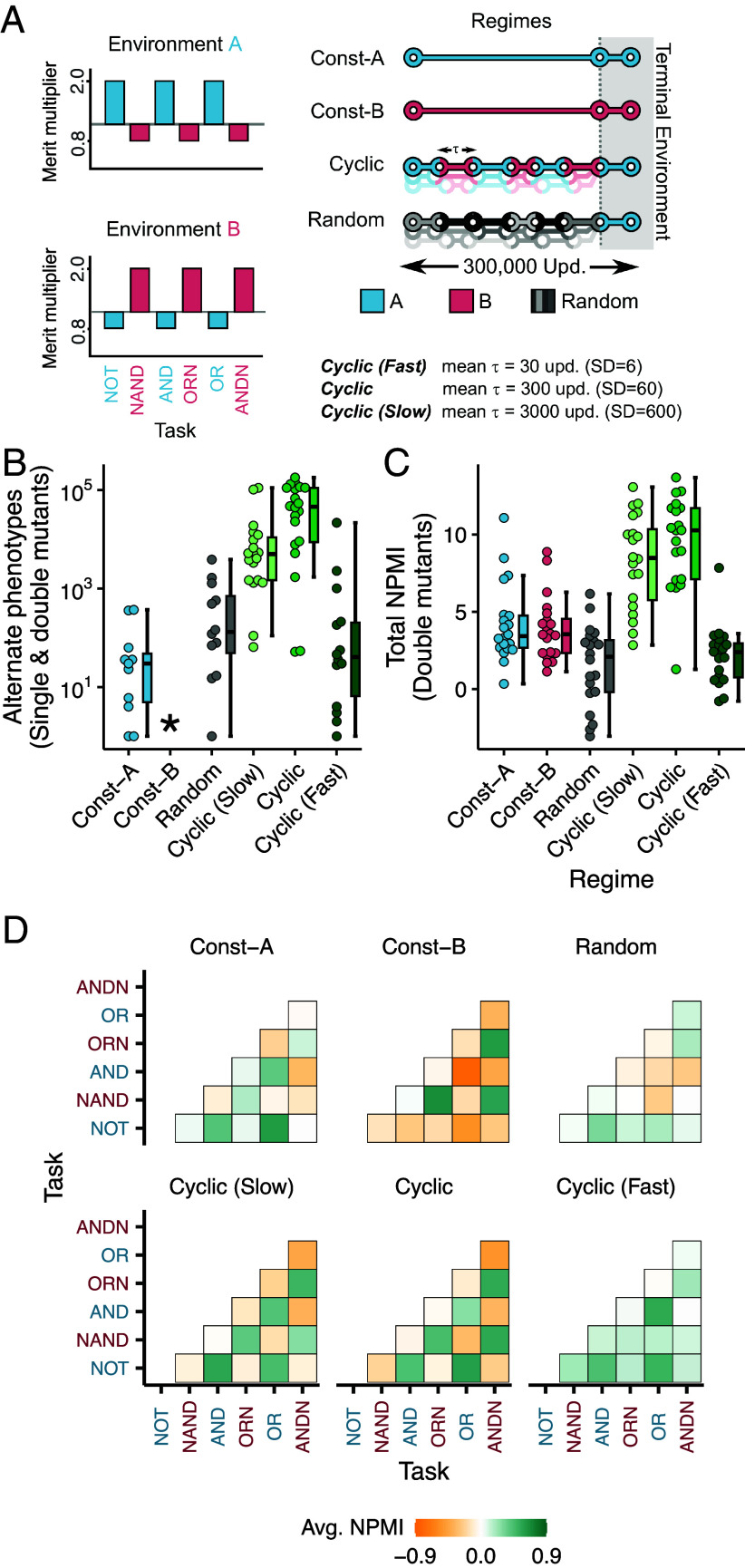
Evolution in changing environment shapes the mutational neighborhood. (*A*) The two environments A and B recognize a set of six tasks performed by the organisms (*Left*, x-axis). The schematic on the *Right* shows the environmental change regimes used in this study and the states they take during evolution. The Cyclic- regimes switch between two environments with an average waiting time, τ (values below figure). The Random regime rewards a randomly picked set of tasks and switches every 300 updates. Each regime ends with 300 updates of evolution in a fixed terminal environment (gray region). (*B*) Total number of single and double mutants of the end-point dominant genotype having an alternate phenotype. (*C*) Total NPMI for the dominant genotypes isolated from the six environmental change regimes, calculated by summing up the individual task-pair NPMIs after assigning them a sign based on their cognate or noncognate nature. (*D*) NPMIs between task pairs for dominant genotypes isolated from populations evolved in different regimes (averaged over 20 replicates). The marginal and joint probabilities were measured using the double mutants of the dominant genotype. Axes labels in blue and red denote tasks rewarded in environments A and B, respectively. The boundaries of the boxplot represent the first ($Q_{1}$) and third quartiles ($Q_{3}$), with the median represented by the central horizontal line. Whiskers extend to maximum values within the upper ($Q_{3}+1.5\times IQR$) and lower fences ($Q_{1}-1.5\times IQR$), where $IQR=Q_{3}-Q_{1}$.

In our experiments, we change between two environmental states—A and B—that reward and penalize an alternate set of tasks. Organisms that perform tasks NOT, AND, or OR are rewarded with a merit multiple of 2.0 in state A and penalized with a multiplier of 0.8 in state B. On the other hand, organisms that perform tasks NAND, ANDN, or OR are rewarded with a merit multiple of 2.0 in state B and penalized with a multiplier of 0.8 in state A (Fig. 2*A*). The environmental states A and B thus have strong fitness trade-offs between traits, with adaptations to environment A being deleterious in environment B, and vice versa.

## Results

### Evolution of the Mutational Neighborhood.

Owing to a many-to-one relationship between genotypes and phenotypes, organisms will differ in their tendency to generate phenotypic variation when mutated (27, 44–46). First, we tested whether the propensity of genotypes to generate mutants with alternate phenotypes can evolve and identified environmental conditions that favored them.

Starting with a naive ancestral genotype, we evolved Avida populations under six different environment change regimes for approximately 30,000 generations (300,000 Avida updates). We fixed the per-site copy mutation rate of the organisms in these experiments at 0.001 with a genome size of 100 instructions, corresponding to, on average, one mutation for every 10 offspring. The Avida world supported a maximum of 22,500 digital organisms. We explored stable environments in two regimes—Const-A, which is always in environment state A, and Const-B, which is always in environment state B. Additionally, four regimes—Cyclic, Cyclic (Slow), Cyclic (Fast), and Random—fluctuate between environmental states. The Cyclic- regimes switch between recurrent environmental states (A→B→A→B...) at different rates. The Cyclic (Slow), Cyclic, and Cyclic (Fast) regimes stochastically switch states every 3, 30, and 300 generations on average, respectively. The Random regime switches randomly between one of 64 possible environmental states every 30 generations on average. Fig. 2*A* shows a schematic detailing these environmental dynamics.

Following experimental evolution, we isolated the dominant sequence (i.e., the genotype present at the highest frequency at the end of evolution) from each evolved population and created all possible single and double point mutations in those sequences (*SI Appendix*, Fig. S1*B*). We then counted the number of mutants with a phenotype that is exactly alternative to the phenotype of the evolved dominant genotype—if the dominant genotype was perfectly adapted to environment A, we counted the number of mutants that were adapted to environment B, and vice versa. Fig. 2*B* shows the number of these mutants with the alternative phenotype that evolved under different regimes (*SI Appendix*, Fig. S3*A* details the overall distribution of all mutant adaptations).

Genotypes isolated from the Cyclic regime had a markedly larger pool of mutants expressing the alternative phenotype when compared to genotypes from constant environments (median for Cyclic=45802.5 versus median for Const−A=1.0; Mann–Whitney U=6, n=20, $P=1.4\times 10^{-7}$, two-tailed. No alternate phenotype mutants were observed for Const-B genotypes). This escalation in alternate phenotypes was contingent on a predictable environment; the Random regime lacked genotypes with increased accessibility of alternate phenotypes (median for Random=16.0 versus median for Const-A =1.0; Mann–Whitney U=224, n=20, p=0.221, two-tailed). Notably, genotypes isolated from the intermediate Cyclic regime—with switches every 30 generations—generated more alternate phenotypes on mutation compared to other regimes (median for Cyclic(Slow)=4556.0, median for Cyclic=45802.5; Mann–Whitney U=312,n=20,P=0.0019, two-tailed). To test whether these alternative phenotypes were enriched in the mutational neighborhood at the expense of robustness, we measured the correlation between the number of four different classes of mutants—alternate (perfectly adapted to the alternate environment), terminal (perfectly adapted to the terminal environment), intermediate (perfectly adapted to neither environments), and inviable (unable to self-replicate). The number of alternate phenotype mutants was not significantly correlated with changes in any other category (*SI Appendix*, Fig. S3*B*). Thus, our results confirm that increased evolvability does not necessarily lead to a reduction in genetic robustness (32, 47).

These results indicate that the populations subjected to cyclically changing environments tend to evolve toward regions in the genotype space where adaptive phenotypes—those with high fitness in frequently encountered environments—are mutationally accessible. To test whether the mutational neighborhoods of evolved genotypes encode measurable information about the historic environment states, we analyzed the normalized pointwise mutual information (NPMI) between tasks. The NPMI metric calculates whether the co-occurrence of any two tasks within a genotype’s mutational neighborhood is significantly higher than expected assuming independent task probabilities (*Materials and Methods*). Fig. 2*D* shows the NPMIs for different task pairs. We observed that the mutational neighborhood of organisms from changing environments systematically captured the pattern of tasks rewarded in different environmental states. Specifically, in Cyclic and Cyclic (Slow) regimes, task pairs that are rewarded concurrently in an environment state (i.e., are cognate) yielded positive NPMIs whereas task pairs that were rewarded in alternate environments had a negative NPMI.

We calculated the total NPMI by consolidating the task-pair NPMIs depending on whether or not the task pairs were cognate in cyclic environments. This allowed a total quantification of information about the environment that was encoded in the mutational neighborhood. Fig. 2*C* shows the total NPMIs. The median total NPMIs for Cyclic and Cyclic (Slow) genotypes were considerably larger than NPMIs from the static regimes (Median for Const-A =3.43, median for Cyclic=10.28; Mann–Whitney U=46, $P=8.17\times 10^{-6}$, two-tailed). Further, to exclude the influence of perfectly adapted mutants, we recalculated the total NPMIs after removing all mutants that were perfectly adapted to the terminal environment (*SI Appendix*, Fig. S4). Even without the contributions from these mutants, the Cyclic and Cyclic (Slow) genotypes had larger total NPMIs (Median NPMI for Const-A =−0.43, Cyclic median = 3.59; Mann–Whitney U=52, $P=2.138\times 10^{-5}$, two-tailed). These results demonstrate a twofold effect of evolution in predictably switching environments: genotypes both had access to more mutants with an alternate phenotype, and their mutational neighborhoods reflected traits in a manner that mimicked the structure of environments the population had encountered in the past.

### Evolution Along Lineages.

**Fig. 3.**
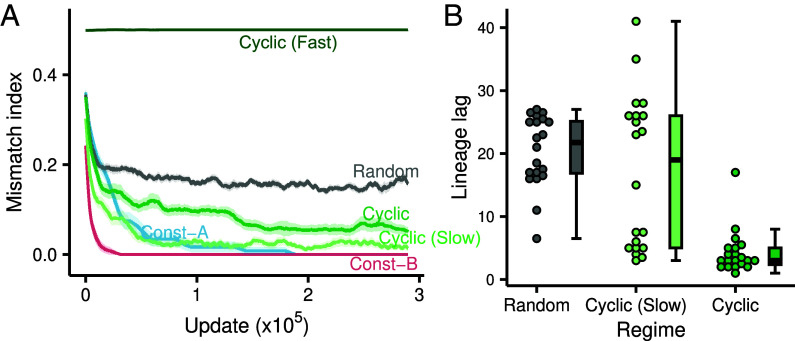
Lineages from Cyclic regime adapt better and faster to their environments (*A*) Moving window average of the mismatch between environment and lineage phenotypes over evolutionary time (window size = 10,000 updates). The mismatch index measures the number of task losses or gains required to be perfectly adapted to the extant environment. Width of the ribbons is SE from 20 replicates. (*B*) Lineage lag between the environment and the lineage phenotypes. Lineage lag is calculated as the number of updates elapsed until the lineage achieves a perfectly adapted phenotype after the environment switches. Only lineages from Random, Cyclic (Slow), and Cyclic regimes are shown here as they consistently achieved perfect adaptation within 100 updates after a switch. Each point is the median lineage lag from different environmental switches over an experiment. Box-plot characteristics same as in Fig. 2.

To investigate the dynamics that shaped the mutational neighborhood, we isolated the lineage of the dominant genotypes all the way to the ancestral genotype, giving us a complete record of the mutations that occurred during evolution. We then calculated the mismatch between the environment state and the phenotypic state of the lineage over evolutionary time (see *Materials and Methods*; Fig. 3*A*). Lineages from Cyclic and Cyclic (Slow) regimes had a lower overall mismatch compared to Random and Cyclic (Fast) regimes (Median mismatch index for Cyclic=0.238, median for Random=0.930; Mann–Whitney U=2, n=20, $P=5.8\times 10^{-11}$, two-tailed). Moreover, Cyclic lineages show a mismatch only about three times that of Cyclic (Slow) even though these lineages evolved in an environment that switched ten times as frequently (Median mismatch index for Cyclic=0.238, median for Cyclic(Slow)=0.072; Mann–Whitney U=353, n=20, $P=9.65\times 10^{-6}$, two-tailed). To estimate the rate of adaptation on the lineage, we analyzed the number of elapsed updates before dominant lineages were adapted to a changed environment—a measure we denote as lineage lag (see *Materials and Methods*; *SI Appendix*, Fig. S1*C*). In comparison to Random or Cyclic (Slow), lineages that evolved under the Cyclic regime exhibited a substantially faster phenotypic response following environmental change (median lineage lag for Cyclic=3.0, median for Cyclic(Slow)=19.0; Mann–Whitney U=50, $P=4.94\times 10^{-5}$, two-tailed; Fig. 3*B*).

**Fig. 4.**
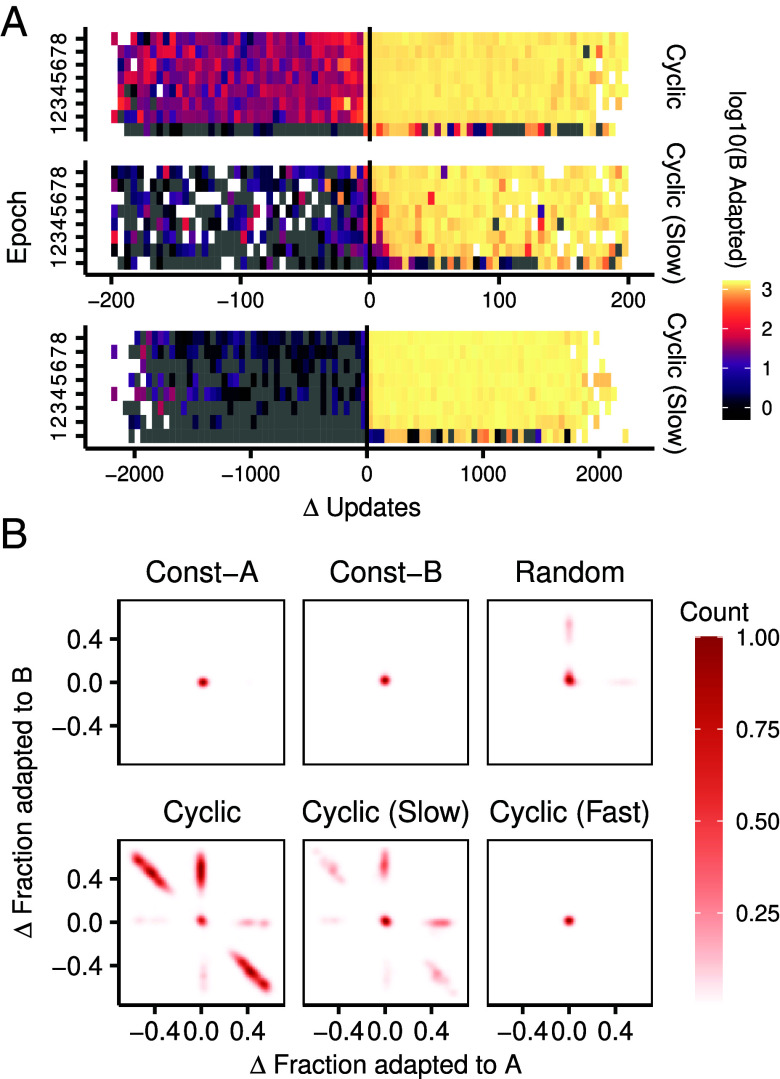
Localization on phenotypic boundaries in the genotype space. (*A*) Median number of lineage sequence mutants that are adapted to environment B before and after the environment switches A→B. The x-axis denotes the number of updates before and after a switch where lineage mutants are recorded. In the top two graphs, each square covers a range of 5 updates. The y-axis segments the entire evolutionary time into eight equal spans, or “epochs.” Only Cyclic and Cyclic (Slow) regimes are plotted here. The lower graph extends the analysis to include up to 2,000 updates around the environmental switch for the Cyclic (Slow) regime, with the range of each square extended to 50 updates. Gray squares denote points where lineages mutate but do not have any B-adapted mutants. White squares denote points where the lineage does not mutate within the time span of observation. (*B*) Trade-offs between mutants on the lineage. The x and y axes plot the concurrent change in number of mutants adapted to A and B during mutation events on a lineage. Points along the y=−x line denote a 1-to-1 trade-off between mutant phenotype distributions whenever the lineage has a mutation (*SI Appendix*, Fig. S1*D*). Plot only includes data from the final 100,000 updates of evolution. Points from 20 replicates are aggregated to calculate the density.

We extended our analyses to the mutational neighborhoods of the sequences on the lineage, specifically near environmental switches. An abridged version of these data is presented in Fig. 4*A* (complete data in *SI Appendix*, Fig. S5). We found that mutational neighborhoods for *Cyclic* lineages appeared to anticipate the environment change; these lineages showed a high concentration of alternate mutants before the environment switched, unlike lineages under the Cyclic (Slow) regime that only developed such adaptations gradually after the switch. Unifying our observations thus far, we hypothesized that the lineages evolved in cyclic environments localized on phenotypic boundaries in the genotype space, allowing them to switch rapidly between alternate phenotypes (Fig. 1*C*). Prior theoretical work suggests that lineages positioned along such boundaries are more competitive in the long run than those further away (29, 48, 49). We tested evolved lineages for this localization by calculating the mutational trade-offs between adjacent sequences along a line of descent. Essentially, we measured the change in the number of mutants adapted to both environments A and B whenever there was a mutation on the lineage (*Materials and Methods*). Lineages that localize on boundaries between well-adapted phenotypes will show the development of strong mutant phenotype trade-offs when comparing temporally adjacent genotypes (*SI Appendix*, Fig. S1*D*). Fig. 4*B* shows these trade-offs as recorded during the last 10,000 generations. Lineages from both Cyclic and Cyclic (Slow) regimes exhibit trade-offs, but Cyclic lineages show the strongest signature. These trade-offs gradually developed as populations evolved and remained stable for long periods of time (*SI Appendix*, Fig. S6).

Next, we tested the evolved populations under alternate rates of change and found that populations from the Cyclic regime adapted faster across fluctuation rates, compared to their counterparts evolved in slower or faster switching environments (*SI Appendix* and *SI Appendix*, Fig. S8). The utility of the evolved mutational neighborhoods thus persisted across fluctuation rates but evolved only under a specific rate of environmental change.

Together, these results demonstrate that selection under changing conditions actively shapes the mutational landscape of populations in a manner that is dependent on the rate and nature of environmental change. Both the accessibility of alternative phenotypes and the information stored in the distribution of mutant phenotypes are substantially enhanced during this process. These features, in turn, help the population switch phenotype quickly when facing ongoing environmental fluctuations.

### Evolution of Mutation Rates.

**Fig. 5.**
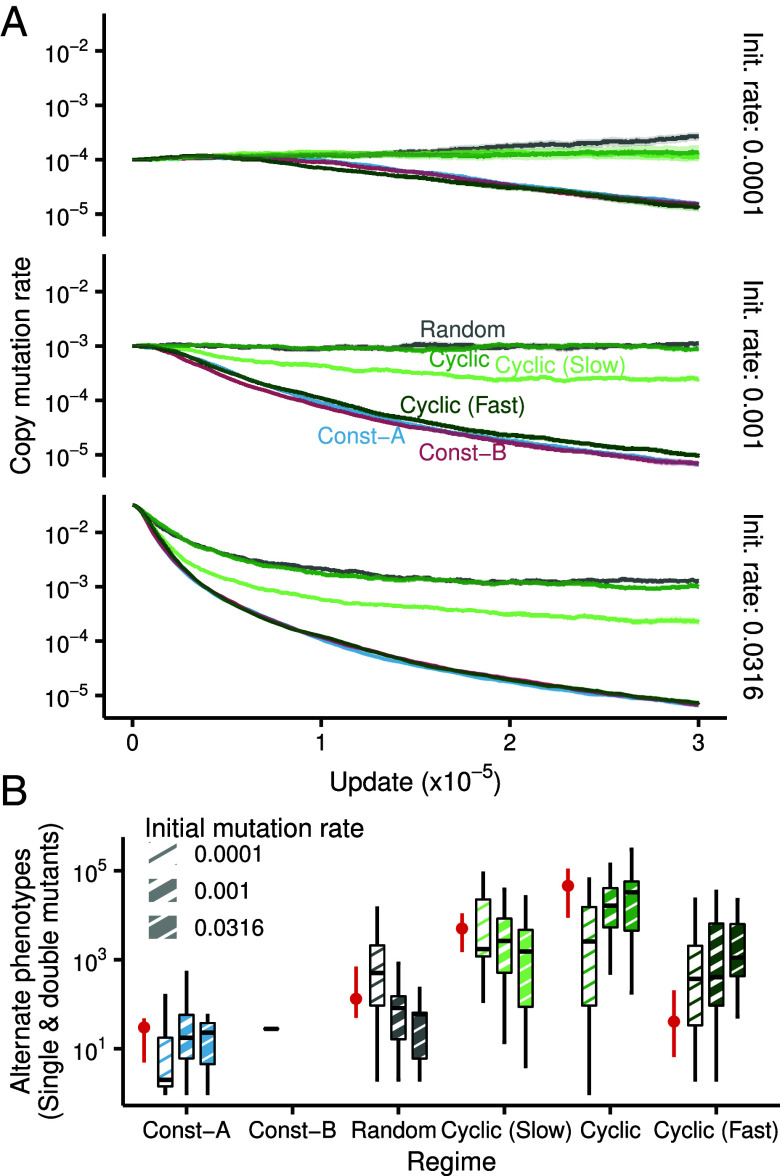
Concurrent evolution of mutation rates and mutational neighborhoods under fluctuations. (*A*) Mean mutation rate for populations evolved under different regimes of environmental change. Center lines represent the average mutation rates calculated from 20 distinct replicates. Ribbons denote 2× SE. The three panels show data from experiments started with different initial mutation rates. (*B*) Total number of single and double mutants of the end-point dominant genotype with an alternative phenotype. The patterned box plots categorize dominant genotypes according to the initial mutation rates used in the experiments. Other box plot characteristics are the same as in Fig. 2. The red filled circles denote the median number of alternate phenotype mutants from the fixed mutation experiments, with the error bars denoting the lower and upper quartiles.

Mutation rates in natural populations are known to evolve rapidly under stressful conditions (50, 51). However, the drift barrier hypothesis proposes that the mutation rate should decrease to a minimal value sustained only by genetic drift (52). To explore the dynamic interaction between mutation rate evolution and the evolution of the mutational neighborhood, we conducted experiments allowing the mutation rates to evolve naturally. In Avida, organisms can inherit their parent’s mutation rate, subject to small stochastic variation (*Materials and Methods* and *SI Appendix*, Fig. S1*E*). To mitigate bias introduced by any specific starting mutation rate, we initiated experiments with rates spanning several orders of magnitude (0.0001, 0.001, and 0.0316). Then, we measured the average mutation rate of organisms in these populations as they evolved under different regimes. The largest starting mutation rate was chosen just below the limit of mutational meltdown (53). Fig. 5*A* shows the average evolved mutation rates for populations starting from three different initial values. Environments undergoing intermediate fluctuations—specifically, the Cyclic and Random regimes—were conducive to the evolution of higher mutation rates (with initial mutation rate of $10^{-3}$, median evolved mutation rate in $\mathrm{Cyclic}=9.19\times 10^{-4}$, median in $\mathrm{Const}-A=7.37\times 10^{-6}$; Mann–Whitney $U=400,\;n=20,\;P=1.45\times 10^{-11}$, two-tailed). This trend toward greater mutation rates in Cyclic held true across all starting mutation rates studied (with an initial mutation rate of $10^{-4}$, median evolved mutation rate for $\mathrm{Cyclic}=1.88\times 10^{-4}$, median for $\mathrm{Const}-A=1.611\times 10^{-5}$; Mann–Whitney U=387, n=20, $P=5.41\times 10^{-9}$, two-tailed).

Elevated mutation rates can increase the mutational load in populations, diminishing the average fitness within a static environment (54). Hence, we anticipated a detrimental impact of high mutation rates on the accessibility of beneficial alternative phenotypes. Counter to this expectation, the Cyclic regime populations maintained access to mutants adapted to alternative environments, while simultaneously evolving high mutation rates (Fig. 5*B*; for initial mutation rate of $10^{-3}$, median number of alternate mutants for Cyclic=16378.5, median for Const−A=0.0, Const−Amean=27.5; Mann–Whitney U=399, n=20, $P=2.86\times 10^{-8}$, two-tailed). The number of alternate mutants in the evolving mutation rate experiments were comparable to the fixed mutation rate case (median number of alternate mutants in Cyclic with fixed mutation rate =45802.5, Cyclic with an initial mutation rate of 0.001 median = 16378.5; Mann–Whitney U=139, n=20, P=0.102, two-tailed). The evolved NPMIs were also similar to the fixed rate treatment (*SI Appendix*, Fig. S7). Thus, populations evolved higher mutation rates and greater accessibility of adaptive variation for alternative environments simultaneously in the Cyclic regime.

### Adaptation in Novel Environments.

To measure the ability of evolved populations to adapt to entirely new environments, we exposed evolved genotypes to an environment with 127 new tasks, distinct from those rewarded in environments A or B. Over 30,000 generations of further evolution, we measured the total number of tasks performed by the descendants of evolved genotypes—i.e., those that had been initially adapted to different environmental regimes. Data reflecting the progression of task acquisition over time are shown in *SI Appendix*, Fig. S8*C*. We found no marked differences in the rate of adaptation for genotypes evolved in different regimes in this new environment (Kruskal–Wallis $\chi^{2}$ on the number of tasks evolved at the end, for fixed mutation rate genotypes = 4.1292, df=5, P=0.531).

By contrast, genotypes from the evolving mutation rate experiments exhibited significant variation in their adaptive success in this new environment (Kruskal–Wallis $\chi^{2}$ for the number of tasks evolved for evolving mutation rate genotypes =94.221, df=5, $P<2.2\times 10^{-16}$). Notably, genotypes from Random and Cyclic regimes that previously underwent mutation rate escalation acquired more functions than the ancestral genotype (median tasks evolved by ancestral genotype=30, median tasks evolved by Cyclic genotypes = 42.3, 18 of 18 samples evolved more tasks than the ancestral median, $P=7.629\times 10^{-6}$, two-tailed one-sample sign test). Inversely, genotypes conditioned to Const-A, Const-B, and Cyclic (Fast) evolved fewer tasks compared to their ancestor (median tasks evolved by Cyclic (Fast) genotypes = 17.9, 19 of 19 samples evolved fewer tasks than the ancestral median, $P=3.815\times 10^{-6}$, two-tailed one-sample sign test).

To test whether mutation rate was the key driver of these observed differences, we transplanted the evolved mutation rates into organisms from the fixed rate experiments. This exchange recapitulated the difference in the rate of adaptation seen between treatments in this new environment (*SI Appendix*, Fig. S8*C*; Kruskal–Wallis $\chi^{2}$ for the number of tasks evolved at the end for fixed mutation rate genotypes transplanted with evolved mutation rates = 95.167, df=5, $P<2.2\times 10^{-16}$). Conversely, when the ancestral mutation rates were transplanted into genotypes from the evolving mutation rate experiment, their rate of adaptation remained largely unchanged (Kruskal–Wallis $\chi^{2}$ for the number of tasks evolved at the end for the evolution mutation rate genotypes transplanted with the ancestral mutation rates = 3.5192, df=5, P=0.6205). These results suggest that while the ability to access alternate phenotypes provides a mechanism to quickly adapt to previously encountered environments, elevated mutation rates (particularly those that evolve under intermediate fluctuating environments) are a primary driver of adaptation to entirely new conditions.

### Interaction between evolution of the mutation rate and the mutational neighborhood.

**Fig. 6.**
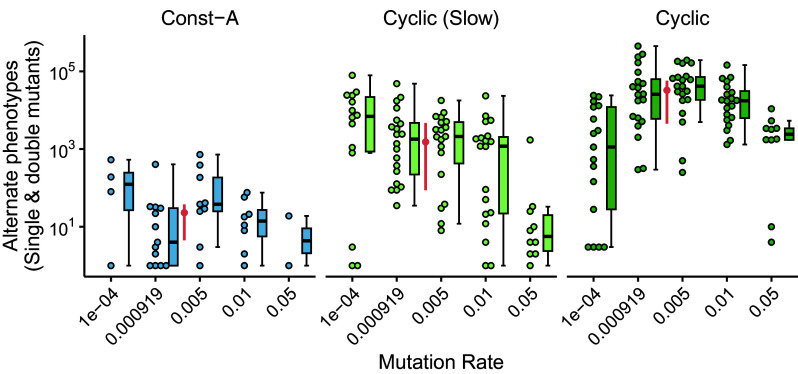
Interaction between mutation rate and environmental change shapes the mutational neighborhood. The y-axis shows the total number of single and double mutants of the end-point dominant genotype that have an alternative phenotype. The data are plotted for evolution experiments in three different regimes with different values of a fixed mutation rate (x-axis). The mutation rate of $9.19\times 10^{-4}$ was set to mimic the evolved mutation rate in previous experiments (specifically, the Cyclic regime with initial mutation rate of $10^{-3}$). The pink circle and error bars denote the mean and quartiles of alternate phenotype counts observed during the evolving mutation rate experiments presented in Fig. 5*A*.

The mutation rate is a key determinant of the rate at which populations explore genetic landscapes and regulates equilibrium genetic diversity (55, 56). Thus, we expect that different mutation rates would lead to distinct evolved mutational neighborhoods. To probe this idea we evolved twenty replicate populations at five different fixed mutation rates in three different environmental regimes (Fig. 6). One of the selected mutation rates mirrored the evolved mutation rate from previous experiments ($\mu =9.19\times 10^{-4}$). We see that the effect of the mutation rate on the evolution of the mutational neighborhood is tied to the way the environments changed. In the static Const-A regime, only a small number of the populations evolved alternate phenotypic mutations. In the Cyclic (Slow) regime, increasing the mutation rate decreased the abundance of alternate phenotypic mutations (P=0.00104; see *SI Appendix*, Table S1). This observation is perhaps expected since frequent mutations would favor populations in more robust regions of the genotype–phenotype map (i.e., “survival of the flattest”) (57). As the mutation rate increased, the drive toward robustness seemed to overpower the evolution of more variable mutational neighborhoods in these slowly shifting environments.

Surprisingly, we found a nonmonotonic yet largely positive correlation between mutation rate and the number of alternate phenotypic mutants in the Cyclic regime ($P=2.39\times 10^{-05}$; see *SI Appendix*, Table S1). The number of alternate phenotypic mutants first increased with mutation rate but subsequently decreased at higher mutation rates (estimated peak at μ=0.0018). We see the largest increase in the number of alternate phenotypic mutants at a mutation rate similar to the one evolved during the original experiments, suggesting the increase in mutation rates may be driven in part by the benefits of reconfiguring the mutational landscape. Considering the number of alternate mutants in Cyclic is always higher than the Slow regime, these results suggest synergy rather than antagonism between the evolution of mutation rates and the availability of alternate phenotype mutations, but only in environments fluctuating at an appropriate speed.

## Discussion

In this work, we experimentally study the simultaneous evolution of the primary determinants of evolvability (the mutational neighborhood and the mutation rate), uncover their interaction with one another, and tie their emergence to the mode of environmental change. We show that mutational neighborhoods can evolve such that organisms have an elevated number of alternate phenotypes that allow them to adapt to changing environments quickly. This occurs when evolving populations move to areas of the genotype space where such phenotype-altering mutations are more abundant—i.e., to phenotype boundaries (Fig. 1*C*). The movement toward phenotypic boundaries is most readily evolved in environments that switch between predictable states at intermediate rates. By predictable, we mean that future environments reliably resemble those encountered previously. In our experiments, the ideal rate for evolvability is realized when the environment switches between two states approximately every 30 generations. Our results indicate that evolution in such environments can favor genotypes that are not necessarily fit presently, but rather have a propensity to generate more adaptive mutants in the future.

We devise a mutual information metric that captures the extent to which the mutational neighborhood reflects the mode of environmental change. Using this metric, we find that the distribution of mutant phenotypes becomes tuned to environments encountered by an evolving population. Thus, the set of phenotypes available to an organism via mutations evolves to store information about environmental change, allowing the organisms to “learn” how to adapt to recurring environments (58). We note an interesting macroevolutionary consequence of this result, where one would expect a lineage that had adapted to changing environments to show greater phenotypic variation than lineages adapted to more stable environments. As a corollary, the variation revealed through mutation can offer clues to the kinds of environments encountered by lineages in the past (38, 59).

Elevated mutation rates can limit adaptation by increasing the accumulation of deleterious mutations (54). Yet, the frequency of such deleterious mutations is contingent upon an organism’s local mutational neighborhood. In agreement with the drift barrier hypothesis, we find that the mutation rate precipitously decreases to minuscule values in static environments or in environments changing extremely rapidly (52). Remarkably, however, we see that the populations maintain much higher mutation rates in environments changing at intermediate rates. In these environments, the value of evolved mutation rates approaches those required for the evolution of the most evolvable mutational neighborhoods (as inferred from static mutation rate experiments). In contrast to previous work where evolution failed to optimize mutation rates in simple environments, we show that such optimization is possible under more complex fluctuating conditions (60). The effect of fluctuations on evolving mutation rates was also observed in a recent study, where populations evolved elevated mutation rates when population bottlenecks were applied at intermediate frequencies during experimental evolution (18). Whether the mutation rate increase observed in our study derives from a similar mechanism-specifically, the pruning of maladaptive phenotypes during environmental shifts-remains an open question.

Counter to the expectation based on compounding effects on the mutational load, we observe increases in both the mutation rate and the accessibility of alternate phenotypic mutants in the recurrent and intermediately switching environments (61). The benefits of these evolvability mechanisms manifest in different ways. On one hand, evolved accessibility of alternate phenotypes helps populations rapidly adapt to previously encountered environments, thus exhibiting a “memory-like” response to changes. Organisms with such evolvability also demonstrate quicker adaptation across a range of environmental change rates, not just the rate they historically experienced. On the other hand, elevated mutation rates allow quick adaptation in completely new environments, where the mutational neighborhood is of little help. Note that the increase in evolvability driven by the mutation rate is a statistical one; higher mutation rates need not lead to improved evolvability in any one specific environment but have a broad adaptive impact when exposed to many environmental challenges. The intermediate-rate environmental fluctuations are also able to overcome the otherwise overwhelming selection for mutational robustness; when the mutation rate is artificially forced high, mutational neighborhoods evolve few alternative phenotypes, except when the environment fluctuates at intermediate rates. Together, adaptation in a predictable intermediate environment primes genotypes for rapid adaptation to both historical and novel environments through modifications to the mutational neighborhood and mutation rate.

The emergence of evolvability in this manner is inherently tied to the structure of genotype space (62–65). Boundaries between distinct regions in genotype space that encode different phenotypes are central to the evolvability that we study, but their presence in every genotype–phenotype map is not guaranteed (66–69). Moreover, such boundaries may not always be accessible through evolution from the ancestral genotype. It is essential to address the abundance of such structural features in the genotype–phenotype map for more general conclusions to be drawn (44, 70). Previously studied models demonstrate that intermediate rates of environmental change influence population localization, but these results are derived from necessarily simple models under idealized assumptions (29, 48). Our work demonstrates that this effect can be readily observed in a realistic–but quite unnatural–evolving system with a markedly complex genotype–phenotype map and identifies the kinds of environmental change regimes that favor such localization.

Our choice of computational model system is not without its limitations. These Avida organisms lack some complex organismal features like multicellularity, sexual reproduction, and an ability to sense their environment. We also configure the environments with strong fitness trade-offs creating selection for mutually exclusive phenotypes, preventing the evolution of generalists that might otherwise succeed (71). In the strictest case, these results may only hold for unicellular asexual organisms with large populations and high mutation rates. For example, in complex organisms phenotypic plasticity could facilitate adjustable phenotypes based on specific environmental cues rather than relying on mutations that only impact future generations (72). However, even such organisms are subject to mutation, selection, and an accessibility of adaptive phenotypes that is dictated by their genotype–phenotype map. Thus, while these effects may be downplayed in more complex organisms, they are still likely to play an important role in their evolution over long timescales.

In conclusion, we show that evolution under specific modes of environmental change promotes high mutation rates and simultaneously shapes the local mutational neighborhood of populations. By measuring the rate of adaptation of evolved organisms in new environments, we show that increases in evolvability acquired through these two mechanisms play distinct roles in historical and novel environments. Using large-scale digital evolution experiments, we trace the dynamics and interplay of these modes of evolvability as they evolve, and show that this escalation is characterized by the movement of populations toward boundaries in genetic space. Future work should aim to analytically resolve the dependence of evolvability on the environment, and test whether our results can be harnessed for the directed evolution of populations with greater evolvability. Although we use an unnatural study system, our results provide insight into how and why populations in nature have been evolving so relentlessly.

## Materials and Methods

### In Silico Experiments.

We initiated all primary evolution experiments with identical ancestral organisms with a genome that was 100 instructions in length and did not perform any tasks. Only substitutions were allowed during mutation. For experiments with a fixed mutation rate, we used a copy mutation rate of 0.001, which created approximately one substitution out of every 1,000 instructions copied. For experiments where mutation rates were allowed to evolve, we started with three different initial mutation rates—0.0001, 0.001, and 0.0316. New offspring inherited the mutation rate of their parents but were modified by adding a deviation drawn from a Gaussian distribution with a mean of 0 and a SD of 1% of the parent’s mutation rate value (*SI Appendix*, Fig. S1*E*) (60).

### Environment Change Regimes.

The Const-A and Const-B regimes were in environmental states A and B, respectively, throughout evolutionary time (Fig. 2*A*). The Cyclic regime switched between environments A and B, with the waiting time between switches picked from a Gaussian distribution with a mean of 300 updates (∼30 generations) and a SD of 60 updates. The Cyclic (Fast) regime switched between A and B with a mean waiting time of 30 updates (∼3 generations) and a SD of 6 updates. The Cyclic (Slow) regime switched between A and B with a mean waiting time of 3,000 updates (∼300 generations) and a SD of 600 updates. In the Random regime, tasks to be rewarded or penalized were redrawn randomly from the set of six tasks described before, with a mean waiting time of 300 updates (SD of 60 updates). All regimes ended with 300 updates of evolution in a constant terminal environment, which was environment B for Const-B, and environment A otherwise. Evolution in these terminal environments allowed us to compare adaptive states at the end of the experiments consistently across regimes.

### Characterizing the Mutational Neighborhood.

We performed an exhaustive survey of the two-step mutational neighborhood of the dominant genotype from the end of the experiments (*SI Appendix*, Fig. S1*B*). To do so, we created every possible single- and double-point mutation in the dominant genotypes isolated after evolution. We tested each mutant for viability (i.e., the ability to self-replicate) and measured the phenotypes for viable mutants (i.e., the set of tasks the mutant could perform). We also isolated the lineages of the dominant genotypes and performed a similar mutational neighborhood analysis on the entire lineage. Only single-point mutations were assayed for lineages. The mutant phenotype data from endpoint dominant genotypes were used to calculate the pointwise mutual information. The mutant phenotype data from lineages were used to measure the mismatch index, lineage lag, and mutational tradeoffs (see below).

### Pointwise Mutual Information.

The pointwise mutual information (PMI) for two events x,y∈Ω is defined as,

[1] $$\begin{array}{c} pmi\left(x,y\right)=\log_{2}\left(\frac{P(x,y)}{p(x)p(y)}\right), \end{array}$$

where Ω is the sample space of events, p is the probability mass function on Ω, and P is the probability mass function on the joint space Ω×Ω. We normalized this value between −1 and 1 by defining the normalized pointwise mutual information (NPMI) as,

[2] $$\begin{array}{c} npmi\left(x,y\right)=\frac{\log_{2}\left(\frac{P(x,y)}{p(x)p(y)}\right)}{-\log_{2}P\left(x,y\right)}. \end{array}$$

The NPMI measures the excess probability of occurrence for certain event pairs in the joint probability distribution when compared to the expected probabilities based on these events’ marginal distribution. For the measurements in this work, x and y denote the different tasks in the set of possible tasks recognized by the environments. p(i) was measured as the fraction of all possible double mutants that performed the task i. P(i,j) was measured as the fraction of all possible double mutants that performed both tasks i and j. A positive NPMI indicates that a task pair is overrepresented in the mutants than expected based on the occurrence of each individual task. A negative NPMI indicates that the task pair is underrepresented on average.

The joint sample space S=Ω×Ω consists of 15 unique, nonsimilar, task pairs. These pairs are labeled as cognate ($\in S_{C}$) or noncognate ($\in S_{N}$) depending on whether they were rewarded by the same environment or not, respectively. The total NPMI was calculated as the sum of individual NPMIs after assigning the task pair a sign based on its cognate/noncognate label.

[3] $$\begin{array}{c} \;\text{Total}\;npmi=\sum_{\left(x,y\right)\in \Omega \times \Omega }s\;npmi\left(x,y\right), \end{array}$$

where,

[4] $$\begin{array}{c} s=\left\{\begin{array}{cc} 1 & ,\;\text{if}\;\left(x,y\right)\in S_{C}\\ -1 & ,\;\text{if}\;\left(x,y\right)\in S_{N}. \end{array}\right. \end{array}$$

We use this total NPMI metric to compare the information encoded on the mutational neighborhood of the dominant genotypes obtained from different regimes.

### Lineage Measurements.

We calculated the mismatch index for lineages as the Hamming distance between the lineage phenotype and the phenotype most fit in the current environment. Hamming distance between phenotypes measured the minimum number of task losses/gains required to change between two phenotypes. For example, an organism that performs NOT, AND, and OR is three units away from an environment that has the maximum fitness for organisms performing NOT and NAND (two losses and one gain of function required). We further averaged the mismatch index over multiple updates to smooth out jumps in the index that happened due to sudden environmental changes.

Lineage lag refers to the time required for the genotypes accumulating along a lineage to become adapted to the environment after it changes (*SI Appendix*, Fig. S1*C*). We surveyed 100 updates both before and after environment switches to see whether the lineage ever acquired a phenotype perfectly fit for the new environment. Then, we calculated the amount of time between when the lineage became perfectly adapted and the environment switch. This number is positive if the lineage switched after the environment changed and negative if it switched before the environment.

To measure mutational trade-offs along the lineage, we calculated the change in the fraction of mutants adapted to environment A (ΔA) and environment B (ΔB) between genotypes occurring along the dominant lineage (*SI Appendix*, Fig. S1*D*). Points with coordinate (ΔA,ΔB) were then plotted in a 2D Cartesian plane. Points along the axes of the 2D plane indicate mutation events along the lineage where an increase or decrease in mutants with one adaptation was not accompanied by a change in the number of mutants with the other adaptation. A high density along the y=−x line denotes a 1-to-1 trade-off between mutations, where an increase in mutants adapted to one environment came at a cost of the number of mutants adapted to the other environment (*SI Appendix*, Fig. S1*D*).

### Cross-Regime Experiments.

We evolved the dominant genotypes—isolated from both fixed and evolving mutation rate experiments—under different regimes to test their performance directly. We released a single organism with the isolated genotype in a new Avida world where the environment switched as described by the four fluctuating regimes—Cyclic, Cyclic (Slow), Cyclic (Fast), and Random. Mutation rates were set based on whether the genotypes were isolated from a fixed or evolving mutation rate experiment but were not allowed to evolve. These Avida worlds had a carrying capacity of 22,500 organisms (same as the primary evolution experiments) and the experiments were run for 10,000 updates. The performance of these genotypes was measured by calculating the time-averaged mismatch and average lineage lag as described earlier.

### Measuring Evolvability in New Environments.

To test the evolvability of organisms in new environments, we released them in an environment where 127 novel tasks—distinct from the six tasks encountered during primary evolution—were rewarded. We then performed a secondary evolution experiment for 300,000 updates where the organisms evolved under a fixed mutation rate. For genotypes isolated from fixed mutation conditions, we used a basal mutation rate of 0.001. For genotypes isolated from evolving mutation rate conditions, the mutation rate was set to the rate they had evolved to during primary evolution. To account for the direct effect of mutation rates, we also performed reciprocal experiments where the organisms that evolved in evolving mutation rates were assigned a mutation rate of 0.001, and the organisms that evolved under fixed mutation rates were assigned the mean mutation rate that evolved in the evolving mutation rate treatments.

### Statistics.

Statistical analyses were performed using functions in base R (73). The correlation plot in *SI Appendix*, Fig. S3 was generated using the corrplot package. Details about the statistical tests, including the type of test used, test statistic value, and p-values are provided in the text. Please refer to the “Plotter/Statistics file path” field in the data guide to obtain the code for the analysis.

## Supplementary Material

Appendix 01 (PDF)

## Data Availability

The data generated for and used in this work have been archived publicly at https://doi.org/10.17605/OSF.IO/7P9KX (74). The data_guide.pdf file therein is a guide that annotates the provided data. The repository contains scripts for experiments, analysis, statistics, and for plotting the figures. Avida, the open-source digital evolution framework used in this work, is available under the MIT license at https://github.com/devosoft/avida (75). All data, scripts, and analysis files used in this work have been archived publicly as mentioned above.
