# Supplementary material for: Evolution takes multiple paths to evolvability when facing environmental change
Source: Proc Natl Acad Sci U S A. 2024 Dec 31;122(1):e2413930121. doi: 10.1073/pnas.2413930121 (PMC11725885; doi:10.1073/pnas.2413930121)
Supplement: Supplementary file 1 — Appendix 01 (PDF) [file pnas.2413930121.sapp.pdf]

Supporting Information for

Evolution takes multiple paths to evolvability when facing environmental change

Bhaskar Kumawat, Alexander Lalejini, Monica Acosta, and Luis Zaman

Corresponding Author: Luis Zaman
E-mail: zamanlh@umich.edu

This PDF file includes:

Supporting text
Figs. S1 to S8
Tables S1 to S2

Supporting Information Text

Other results

Adaptive performance under alternative fluctuation rates. Populations typically excelled when subjected to the same environmental conditions that shaped them; however, it was unclear if such specific adaptations confer benefits across varying ecological conditions. To ascertain whether adaptation within a certain regime delivered cross-regime fitness improvements, we isolated genotypes from the initial evolution experiments and subjected them to each alternative regime. Due to computational constraints, the evolving mutation rate treatment was represented by genotypes from the intermediate initial mutation rate treatment only, with a starting mutation rate of 0.001. To measure the evolutionary performance of genotypes, we calculated the average mismatch index and the average lineage lag over approximately 1,000 generations of evolution. The data is summarized in figure S8A and B.

We found that genotypes evolved in the *Cyclic* regime had a lower average mismatch and adapted faster following environmental changes than their counterparts from the *Cyclic (Slow)* or *Cyclic* regimes. Contrary to expectations, genotypes from variable mutation rate experiments perform worse those from the fixed mutation rate experiments. This decrease in performance may be attributable to the slight reduction in the evolved mutation rates compared to the fixed rates (basal mutation rate=10^{-3}, median evolved mutation rate in *Cyclic* with intermediate starting mutation rate=9.19×10^{-4}). Nevertheless, the possibility remained that adaptation via mutation rates may come at the cost of evolvability via the mutational neighborhood — a hypothesis we examine final section of results.

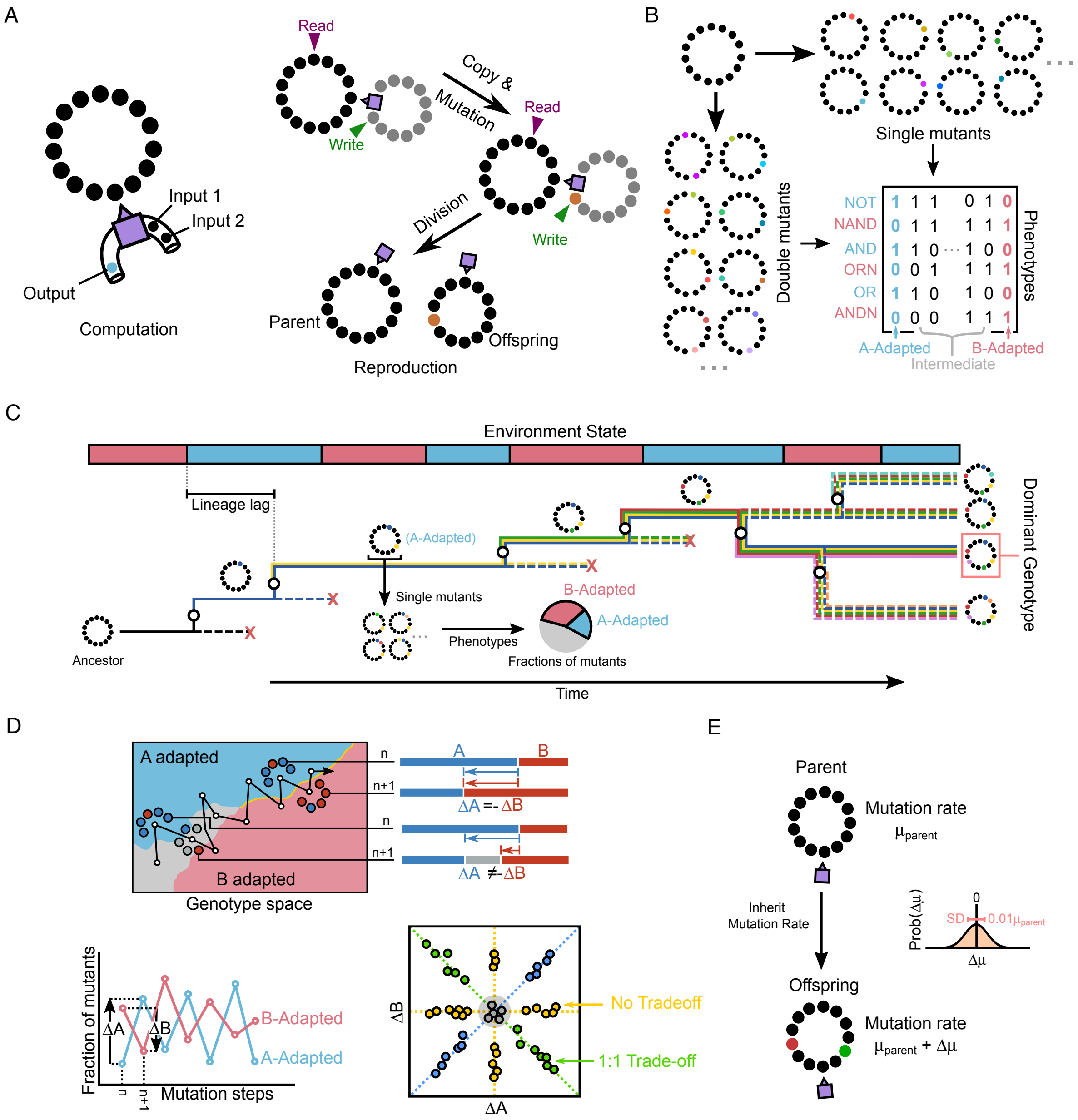

Fig. S1. In silico evolution in Avida and experimental methods. (A) An Avida world consists of multiple genetic sequences, encoded as a circular series of instructions (black circles) that can be executed by a virtual CPU (purple square) that translates these instructions into organismal function. The main organismal functions include *computation* - where organisms perform logical tasks to increase reproductive efficacy - and *reproduction* - where the sequence instructs the CPU to copy instructions into a new memory location (grey and red circles). During computation, the sequence requests inputs from the environment and acts upon these inputs to generate an output. We use two environments in this study - A and B - both of which recognize a set of six possible computations or tasks. Refer to Fig. 2A for more details about the environment states and regimes. (B) To survey the mutational neighborhood of evolved organisms, we create every possible single (top-right) and double mutant (bottom-left) and measure their phenotype. Phenotypes denoting tasks that are rewarded in environments A or B are labeled *A-adapted* or *B-adapted* respectively. All other phenotypes are classified as *intermediate*. Sequences that cannot self-replicate are classified as an *inviable* phenotype (C) An example of a pruned phylogeny of digital organisms evolving in a cyclically changing environment. The colors on a branch denote the set of mutations acquired by the branch compared to the ancestral genome. For most of the data, we look at the lineage of the dominant genotype which is shown as solid lines. Circles on the phylogeny denote mutation events where the phenotype of the lineage may or may not change. *Lineage lag* is calculated as the time it takes for the lineage to adopt a perfectly adapted phenotype following an environment switch, as shown on the top left. When calculating mutational neighborhoods for the lineages, we only look at the single mutants of the lineage genotypes. (D) A hypothesized mechanism of localization that promotes increased access to alternate phenotypes (top). Lineages start in the intermediate adapted region of a genotype space (grey) but slowly find regions where a few mutations allow quick changes in phenotype (yellow curve). If this hypothesis holds, we expect the development of increased mutational trade-offs between consecutive mutational steps on the lineage—i.e., an increase in lineage mutations where the increase in number of mutants adapted to environment A is accompanied by an equivalent decrease in the number of mutants adapted to environment B. We check for these trade-offs by measuring the number of mutants of lineage sequences adapted to environments A and B (bottom-left) and calculating the differences (ΔA and ΔB). When plotted on a Cartesian plane, mutation events that show trade-offs appear along the $y = -x$ line. (E) Schematic of the mechanism for evolving mutation rates. Offspring inherit the mutation rate by sampling from a Gaussian distribution with a mean that is equal to the parent's mutation rate (μ_{parent}) and a standard deviation that is 1% the parent's mutation rate ($\Delta\mu = 0.01\mu_{\text{parent}}$).

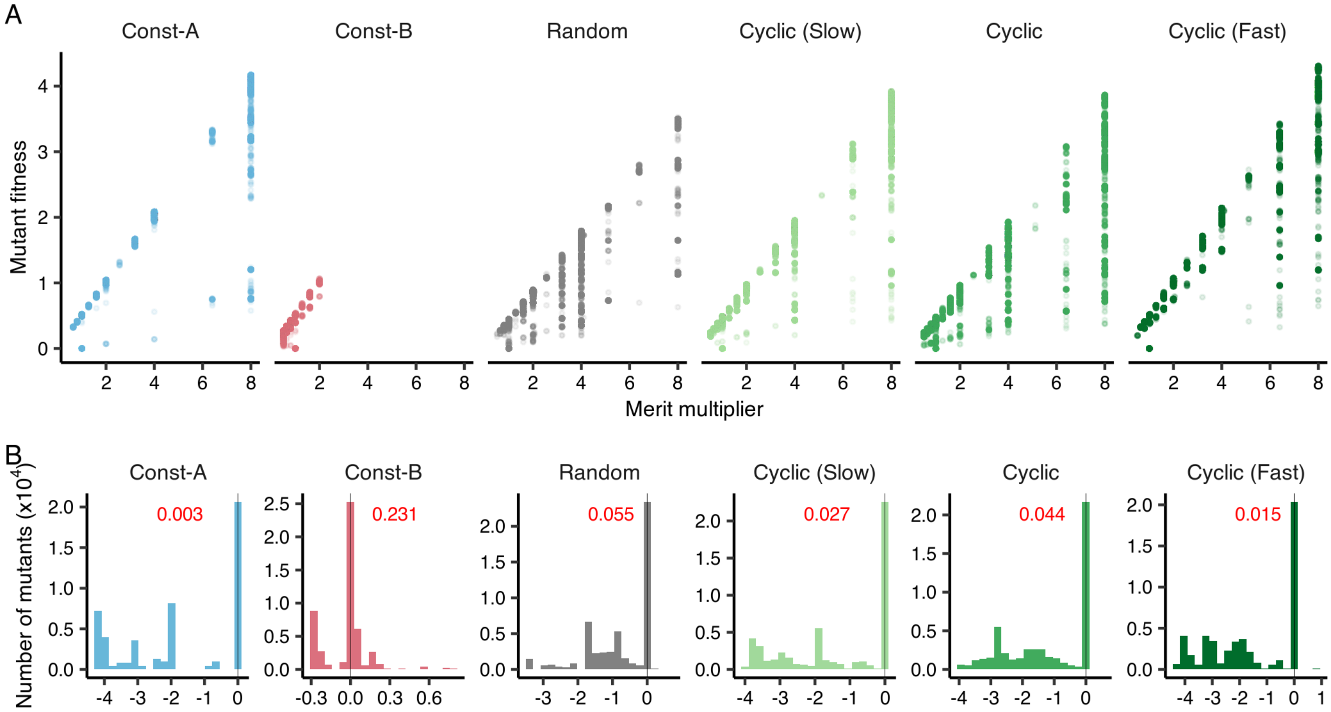

Fig. S2. Merit and fitness. (A) Fitness as a function of the merit for point mutants of dominant genotypes from different regimes. Fitness is calculated as the replication rate in environment state A relative to the ancestral genotype. As shown here, fitness is bounded by the merit but organisms with the same merit may have different fitness values due to mutations in their replication machinery. (B) Distribution of fitness effects (DFEs) of all point mutations to the dominant genotypes isolated from different regimes. The x-axis is the fitness difference between a mutant and its original sequence. The y-axis is the number of mutants with a given fitness difference, derived from all 20 replicates under a regime. Inviable mutants (zero fitness) are included in the leftmost bar in the plot. The red numbers show the fraction of all mutants that are adaptive (i.e., their fitness difference > 0). As seen here, only a small fraction of point mutations are truly adaptive, with most mutations decreasing fitness. The *Const-B* regime is an outlier because evolution in this regime ends in environment B which has strong fitness trade-offs with environment A. Thus, a large number of point mutations are able to break a deleterious task and increase fitness.

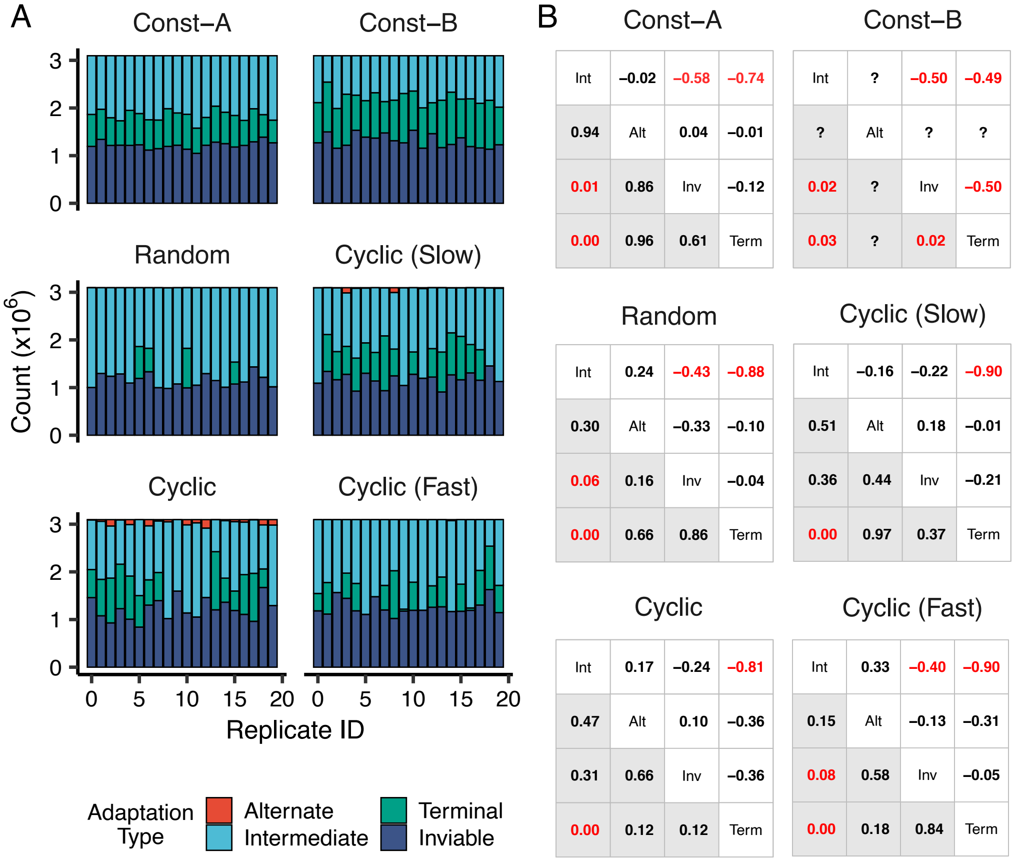

Fig. S3. Distribution of different adaptations in the mutational neighborhood of dominant genotypes. (A) The number of mutants of the dominant genotype - isolated from populations evolved in the different regimes - that are either perfectly adapted to the terminal environment ('Terminal'), the environment alternate to the terminal ('Alternate'), have an intermediate phenotype ('Intermediate') or are unable to self-replicate ('Inviable'). Each bar corresponds to one dominant genotype from a replicate population evolved under a particular environment change regime. Adaptations for both single and double mutants have been plotted here. (B) Correlation matrix between the number of mutants with different adaptation types for dominant genotypes from the six regimes. The upper diagonal numbers denote the Pearson correlation coefficient between the number of mutants with given adaptation types. The lower diagonal numbers (grey) denote the respective p-values for these correlations. Significant p-values (< 0.05) and respective correlation coefficients have been colored red. Text on the diagonal indicates the correlation being measured (Int=Intermediate, Alt=Alternate, Inv=Inviable, Term=Terminal). No alternate adapted mutants were found for organisms from *Const-B* and the values have been replaced with "?".

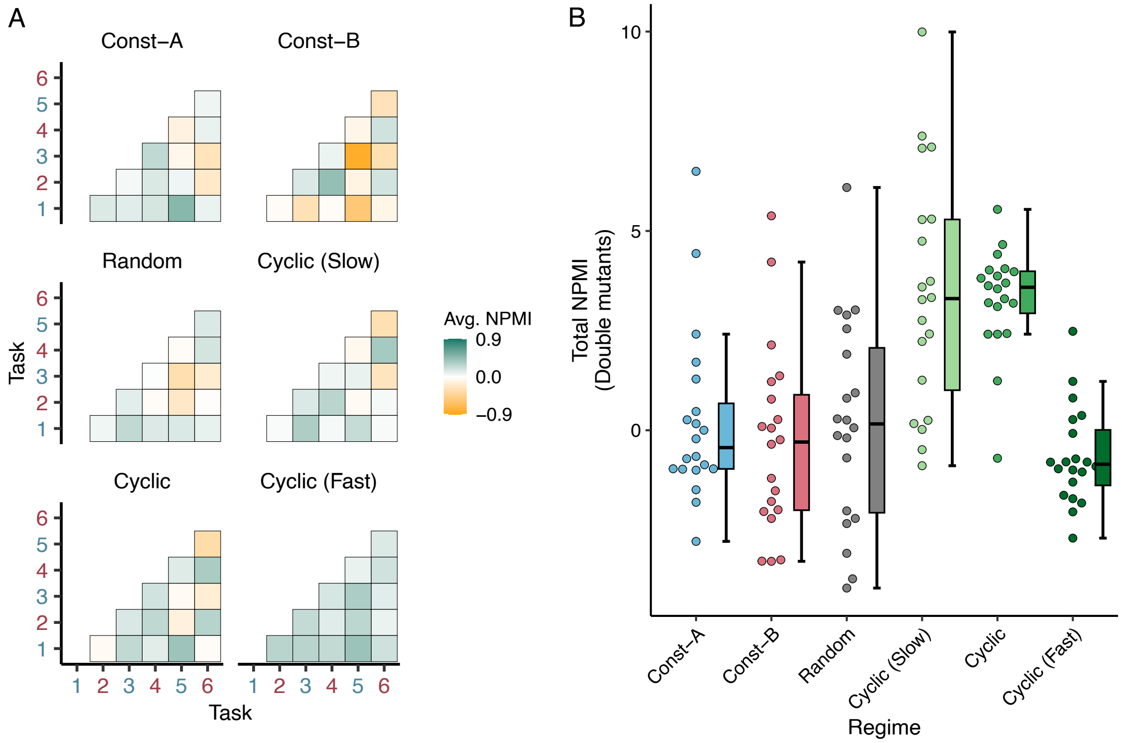

Fig. S4. NPMIs evolved under fixed mutation rate experiments after removal of perfectly adapted mutants (A) Pairwise NPMI between different task pairs plotted using double mutant data. Mutants that are perfectly adapted to environment A or B have been removed. Axes labels in blue and red denote tasks rewarded in environments A and B, respectively. (B) Total NPMIs calculated using the pairwise NPMIs in panel A. Total NPMIs are calculated by summing up the individual task-pair NPMIs after assigning them a positive or negative sign based on their cognate or non-cognate nature. Box-plot characteristics are the same as in Fig. 2.

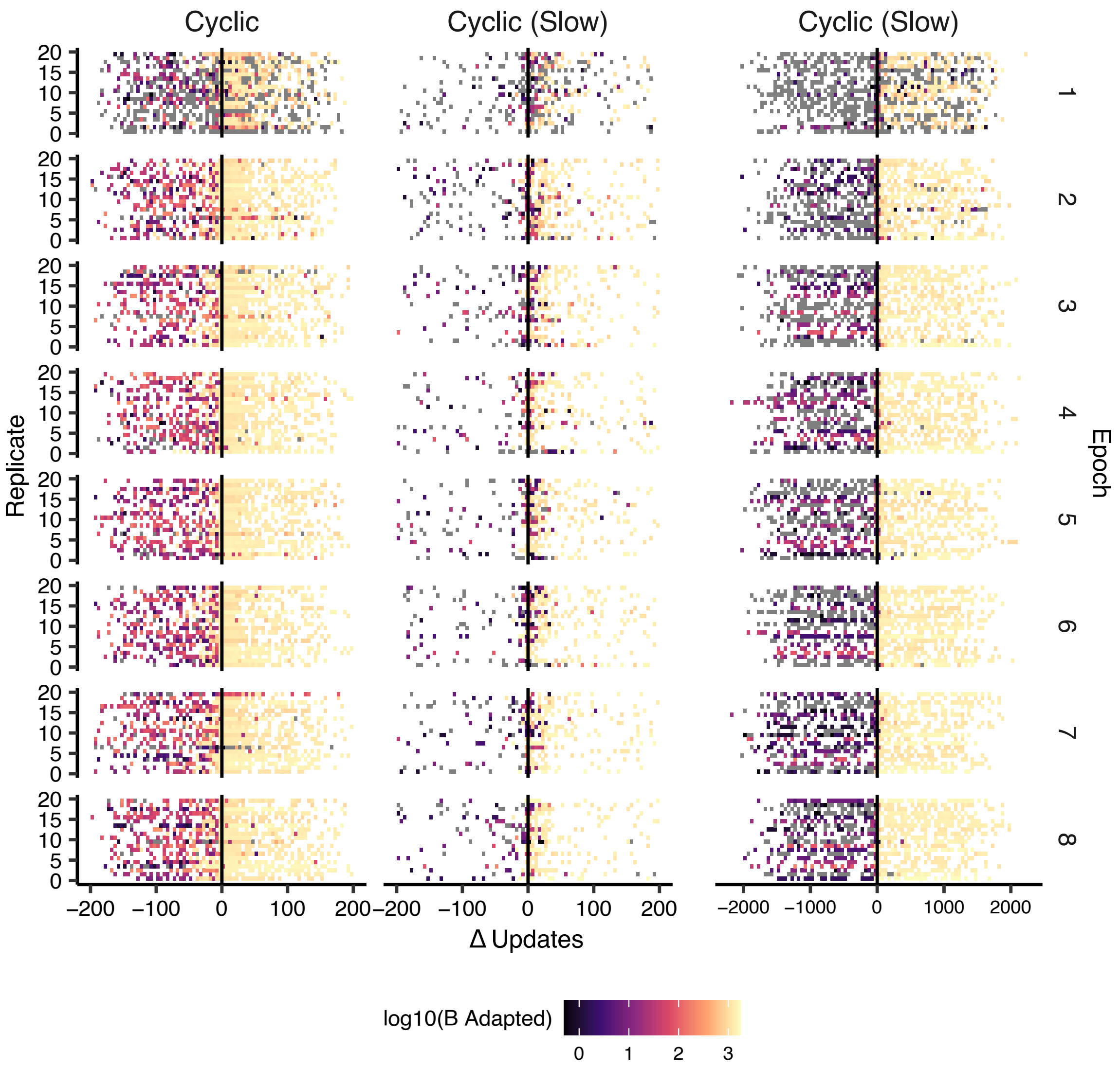

Fig. S5. Median number of point-mutants of lineage sequences adapted to environment B before and after the environment switches from state A to B. The x-axis tracks the number of updates from the point of environmental transition where lineage mutants are recorded. The experiment time has been divided into eight epochs of 37,500 updates (approx. 375 generations) each, shown here as different rows. The y-axis in each figure shows 20 different replicates. Median tallies of point mutants were computed within intervals: bins of 5 updates for the two leftmost graphs, and an expanded bin size of 50 updates for the graph on the far right. The middle *Cyclic (Slow)* figure restricts the range to ± 200 updates.

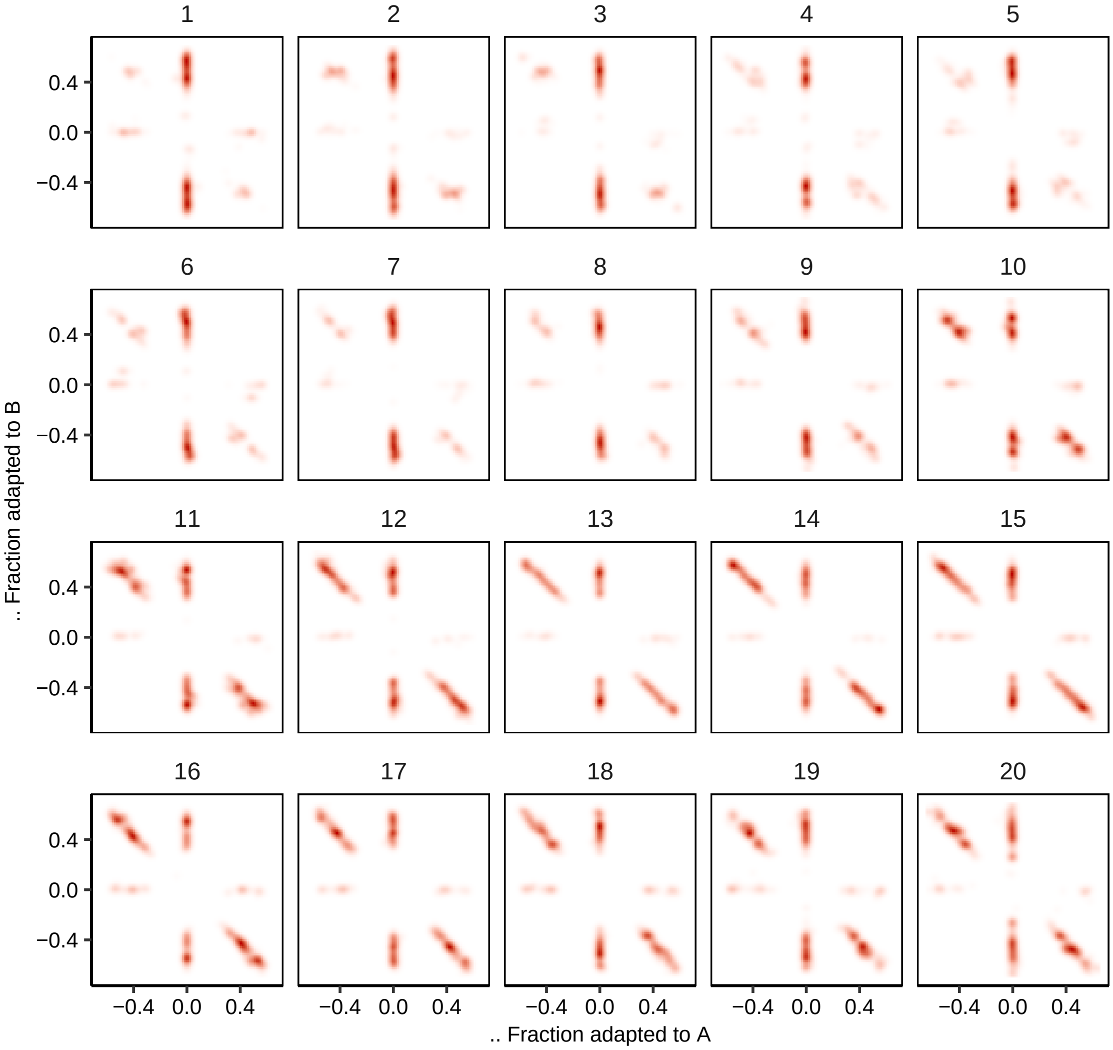

Fig. S6. Mutational trade-offs over evolutionary time under the Cyclic regime. Density of points in the *trade-off plane* (See figure S1D and methods) plotted over 20 equally spaced time intervals during evolution in the *Cyclic* regime (all replicates). Each panel is a time interval spanning 15000 updates (approx. 1500 generations). Points along the $y = -x$ diagonal represent mutation events on a lineage where an increase in number of mutants adapted to a particular environmental state was accompanied by an equal decrease in the number of mutants to the alternate environmental state.

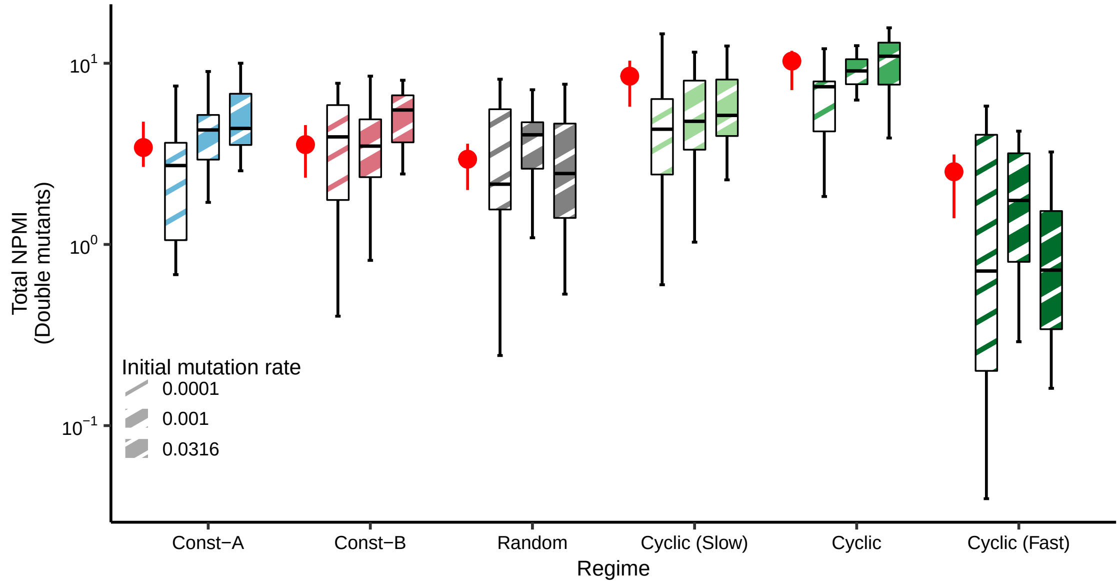

Fig. S7. Evolved total NPMIs under fixed and evolving mutation rates. Total NPMI for the dominant genotypes isolated from the six environmental change regimes with evolving mutation rates. Box plots with different patterns denote NPMI values from experiments that started with different initial mutation rates. The red filled circles denote the median NPMI for genotypes from the fixed mutation rate experiments, with the error bars denoting the lower and upper quartiles.

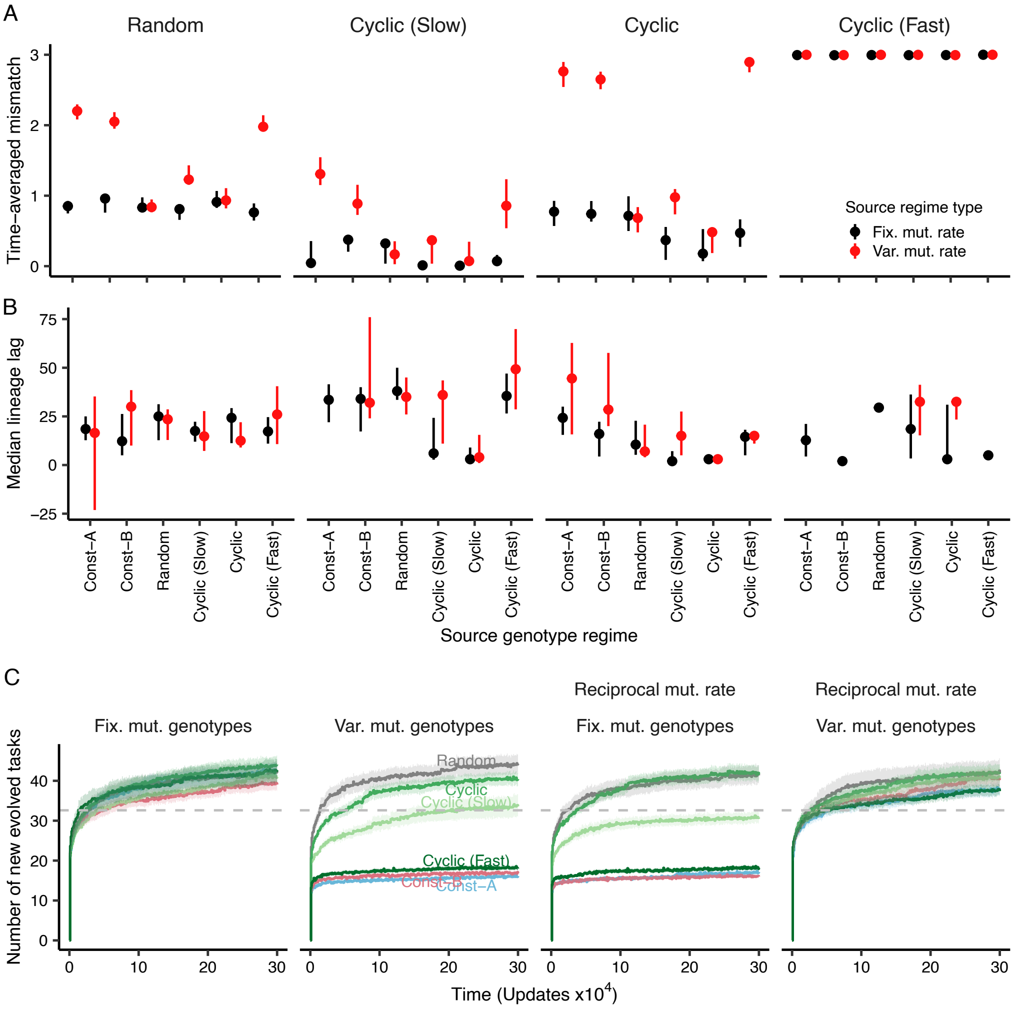

Fig. S8. Performance of evolved genotypes transplanted into other regimes and completely new environments. (A) Median value of the time-averaged mismatch (over 10,000 updates) for genotypes isolated from primary evolution experiments in different test regimes (columns). The mismatch is calculated as the hamming distance between the dominant lineage phenotype and the environmental state—i.e, the number of task gains/losses required by the phenotype to be perfectly adapted to the extant environment. The x-axis denotes the regime the genotype was isolated from. (B) The median value of the mean lineage lag for genotypes from primary evolution in different test regimes. The lineage lag measures the time taken by the lineages to achieve a phenotype perfectly adapted to the environment after a switch. (C) The number of tasks evolved by genotypes from different source regimes when evolving in a new environment with 127 new tasks that increase fitness incrementally. The first panel shows the evolution of tasks in this new environment for genotypes isolated from the fixed mutation rate experiments (with a mutation rate of 10^{-3}). The second panel shows the evolution of genotypes from evolving mutation rate experiments, retaining their evolved mutation rates. The third panel shows the evolution of fixed mutation rate genotypes when assigned the average evolved mutation rate from the evolving mutation rate experiments. The fourth panel shows the evolution of genotypes from evolving mutation rate experiments when assigned a basal mutation rate of 10^{-3}. The dark lines denote the average across 20 replicates, and the ribbons represent the standard error.

Table S1. Example of an `AND` logical task encoded as a series of 9 consecutive instructions in an Avida genome. Each Avida organism has three 8-bit registers (A, B, and C) and an infinite stack. Instructions can be used to move values between these components, or to move them to and fro from the Avida environment. A description of what each instruction does in this context is provided. This hand-written version is optimized and relies on the logical formula `X AND Y = (X NAND Y) NAND (X NAND Y)`. An actual Avida organism may use more instructions than this to perform the same task (and even use them in a non-contiguous manner).

#	Instruction	Instruction Description	Register Contents after instr.			Stack Contents	Output
			A	B	C		
1	IO	Output contents of B and get input (X) from the environment into B	?	X	?	?	?
2	IO	Output contents of C and get input (Y) from the environment into C	?	X	Y	?	?
3	nop-C						
4	nand	Perform (B NAND C) and store it in register B	?	X NAND Y	Y	?	
5	push	Copy contents of register B into the stack	?	X NAND Y	Y	X NAND Y, ?	
6	pop	Copy top value in stack to register C	?	X NAND Y	X NAND Y	?	
7	nop-C						
8	nand	Perform (B NAND C) and store it in register B	?	X AND Y	X NAND Y	?	
9	IO	Output contents of B and get input (Z) from the environment into B	?	Z	X NAND Y	?	X AND Y

Table S2. Statistical analyses associated with the number of alternate mutants evolved under different static mutation rates.

Regime	Model	BIC	Estimate
Cyclic (Slow)	num_alternate ∼a * mutrate + b	2145.591	
	num_alternate ∼a * mutrate^2 + b * mutrate + c	2146.862	
	num_alternate ∼a * log(mutrate) + b	**2140.682**	a = -3718 (p=0.00104)<br>b = -4900 (p=0.10096)
	num_alternate ∼a * log(mutrate)^2 + b * log(mutrate) + c	2145.264	
Cyclic	num_alternate ∼a * mutrate + b	2514.165	
	num_alternate ∼a * mutrate^2 + b * mutrate + c	2518.596	
	num_alternate ∼a * log(mutrate) + b	2520.119	
	num_alternate ∼a * log(mutrate)^2 + b * log(mutrate) + c	**2506.231**	a = -35136 (p=2.39e-05)<br>b = -192056 (p=2.15e-05)<br>c = -196493 (p=0.0004)
